# Batson’s plexus and retrograde venous spread of malignancy – a pictorial review

**DOI:** 10.1186/1470-7330-14-S1-P40

**Published:** 2014-10-09

**Authors:** RC Brook, K Tung, R Oeppen

**Affiliations:** 1 Department of Clinical Radiology, Southampton University Hospital, Southampton, UK

## Aim

Batson’s venous plexus is a system of paravertebral veins that connect pelvic and thoracic vessels to the intraspinal (basivertebral) veins. It was first described in 1940 to explain a route for spread of metastases and infection that was separate to the lymphatic system. Its role in the retrograde venous spread of malignancy is now well-described, but not widely demonstrated on imaging.

## Method & results

We present a detailed pictorial review of imaging of patients from our oncology centre showing retrograde venous spread to the paravertebral vessels specifically in cases of renal, rectal and breast carcinoma.

We demonstrate expansion of the paravertebral vessels containing tumour and associated vertebral body metastases.

We also review and illustrate the spinal venous anatomy.

## Conclusion

Radiologists should be aware of the implication of Batson’s venous plexus as a route of metastatic dissemination. Our pictorial review highlights the importance of the paravertebral vessels as a review area.

